# Self-Optimization in Continuous-Time Recurrent Neural Networks

**DOI:** 10.3389/frobt.2018.00096

**Published:** 2018-08-21

**Authors:** Mario Zarco, Tom Froese

**Affiliations:** ^1^Departamento de Ciencias de la Computación, Instituto de Investigaciones en Matemáticas Aplicadas y en Sistemas, Universidad Nacional Autónoma de México, Mexico City, Mexico; ^2^Centro de Ciencias de la Complejidad, Universidad Nacional Autónoma de México, Mexico City, Mexico

**Keywords:** modeling, optimization, Hopfield neural network, Hebbian learning, fixed-point attractors

## Abstract

A recent advance in complex adaptive systems has revealed a new unsupervised learning technique called self-modeling or self-optimization. Basically, a complex network that can form an associative memory of the state configurations of the attractors on which it converges will optimize its structure: it will spontaneously generalize over these typically suboptimal attractors and thereby also reinforce more optimal attractors—even if these better solutions are normally so hard to find that they have never been previously visited. Ideally, after sufficient self-optimization the most optimal attractor dominates the state space, and the network will converge on it from any initial condition. This technique has been applied to social networks, gene regulatory networks, and neural networks, but its application to less restricted neural controllers, as typically used in evolutionary robotics, has not yet been attempted. Here we show for the first time that the self-optimization process can be implemented in a continuous-time recurrent neural network with asymmetrical connections. We discuss several open challenges that must still be addressed before this technique could be applied in actual robotic scenarios.

## Introduction

Unsupervised learning techniques have many applications, especially to complex problems that we would like to be solved automatically, but without already knowing what the correct responses are to begin with. One such area of application is cognitive robotics: although we can prepare the cognitive architectures of the robots by design and/or artificial evolution to some extent, real-world scenarios always involve unexpected changes and events that a robot should ideally be able to learn to adapt to spontaneously. One popular approach is self-modeling, for example a multi-legged robot that adapts its controller to its physical body by evaluating its sensory feedback against an internal simulation of its possible body morphology and how that body would interact with its environment (Bongard et al., 2006). Another approach, which avoids the use of an explicit internal model, is homeostatic adaptation: for example, a multi-legged robot with a homeostatic neural controller that will cycle through structural changes to the neural network until a motion pattern is found that permits it to maintain the neural activation states within the homeostatic range (Iizuka et al., 2015).

Both of these approaches have their advantages and disadvantages. The use of an internal model permits more control over how the robot responds to challenges, but it therefore ultimately relies on explicit knowledge and detailed representations of the kinds of challenges that could be faced by the robot. The homeostatic mechanism remains agnostic about the kinds of challenges that could push it outside of its region of stability, and is therefore potentially more robust, but its way of recovering internal stability may not always involve recovery of the original desired behavior because there are usually several ways of satisfying internal homeostatic constraints (Di Paolo, 2003). It also has the disadvantage that it is defined only negatively: something in the robot has to break down before the homeostatic mechanism springs to action and starts changing the connection weights of neurons until they recover stability, for example by applying Hebbian learning (Di Paolo, 2000).

Our aim in this article is to investigate the possibility of an unsupervised learning technique that retains the advantages of this implicit approach to structural adaptation, but which might be able to address some of its shortcomings by turning the homeostatic mechanism upside down: instead of modifying connections only when the neural controller is in an undesirable state configuration until it finally leaves that configuration, it may be more fruitful to apply Hebbian reinforcement precisely during moments of stability and to repeatedly force the neural controller into unstable regions of state space in order to enable it to converge on and learn about different stable configurations. In other words, could we use principles similar to homeostatic adaptation but in a way that permits the neural controller to self-optimize its structure in a more directed, memory-dependent manner?

A suitable starting point for this endeavor is a discovery in the area of complex adaptive systems made by Watson et al. (2011b), who found a simple technique that permits a Hopfield neural network to spontaneously optimize its own connection weights so as to become better at satisfying the constraints that were specified by the network's original weight configuration. They showed that in this way the network will converge on a configuration that will permit it to find the best solutions to the original problem space from any initial condition, even if these solutions would have been practically impossible to find before the application of the technique. The key to this apparent magic is the combination of two capacities of the Hopfield network that are normally studied separately: associative memory formation and constraint satisfaction. The network is allowed to repeatedly converge on suboptimal attractors, which are then reinforced by Hebbian learning, with the result that the network starts to form an associative memory of the visited attractors, which permits it to generalize to finding new, better attractors. Although Watson and colleagues describe this process as self-modeling, we prefer the term *self-optimization* because the technique does not involve any explicit internal model.

We are interested in whether this process of self-optimization can be applied in evolutionary robotics as a form of unsupervised online learning to generate more adaptive behaviors. Many tasks require smooth behavior that depends on the fine-grained temporal dynamics of the neural controller, which is why agent-based modeling of adaptive behavior typically relies on a continuous-time continuous-state neural network (Beer, 1997, 2008), such as the widely studied continuous-time recurrent neural network (CTRNN). Moreover, unlike the structure of a Hopfield network, in most cases the CTRNNs used in these tasks are not restricted to symmetrical weights and neurons without self-connections. In other words, even when leaving aside the open challenges of how to implement self-optimization in a neural controller that is coupled to a body and an environment, it is currently not even clear how (or if at all) the self-optimization process could operate in a decoupled CTRNN under these dynamically much richer conditions.

Thus, our immediate aim in this article is to demonstrate that self-optimization can be realized in an unconstrained, albeit decoupled, CTRNN. We find that this process spontaneously restructures the connection weights such that the CTRNN's attractor landscape tends to become dominated by an attractor that on average resolves more interneuron constraints. This opens the door for future work on how to implement self-optimization in coupled, brain-body-environment systems.

At first sight it may seem rather limiting that the self-optimization process consistently results in global fixed-point attractors, but it has been demonstrated that monostable controllers are not limited to single behaviors: they are capable of exhibiting distinct adaptive behaviors by taking advantage of the complex folds of their single basin of attraction (Buckley et al., 2008). Moreover, when an evolutionary robotics approach is employed to generate agent-based models of adaptive behaviors in different task domains, it is frequently found that the most flexible evolved solutions involve neural controllers with only single attractors; however, the location of this attractor and the shape of its basin of attraction are transformed appropriately via the agent's coupling with its environment (e.g., Froese and Fuchs, 2012; Buhrmann et al., 2013; Agmon and Beer, 2014; Campos and Froese, 2017). In all of these models the appropriate network structure is evolved and remains fixed, and it would be an important advance to find a mechanism that allows that structure to be learned and to be open to further plastic changes according to online changes in task demands. At the end of the article we will consider the open challenges that remain to be addressed in order to extend our promising findings with decoupled CTRNNs to these kinds of “world-involving” (Di Paolo et al., 2017) scenarios.

The rest of this article is structured as follows. In section Hopfield Neural Networks we briefly summarize the two main applications of Hopfield neural networks, which form the basis of self-optimization. In section A Review of Self-Optimization in Neural Networks we review existing work on self-optimization in neural networks, which largely remains within the classical formalism of the Hopfield neural network. In section Methods we introduce our own approach, which generalizes the self-optimization mechanism to a CTRNN. In section Results we present the results of our study, and in section Discussion we conclude by means of a discussion.

## Hopfield neural networks

The Hopfield neural network (HNN) was first introduced by Little (1974) and then popularized by J. J. Hopfield. In the literature, three main types of Hopfield neural networks can be found depending on the type of activation function and the temporal dynamic: discrete-time discrete-state (Hopfield, 1982), discrete-time continuous-state (Koiran, 1994), and continuous-time continuous-state (Hopfield, 1984). In general, these neural network models have been used mostly for associative memory formation (Hopfield, 1982) and optimization problems resolution (Hopfield and Tank, 1985). We will describe these two main applications in more detail because together they form the basis of the self-optimization process.

Associative memory formation has two phases: learning and recall. The learning phase is based on a set of training patterns using a learning rule which updates the weights of the network so as to form fixed-point attractors (hereinafter referred to as attractors). These attractors correspond to the patterns learned by the network, even though there exist spurious attractors which do not correspond to any of the training patterns. In the recall phase, a test pattern, usually an incomplete or noisy version of a stored pattern, is set as the initial state of the network. The states of the network are then updated until the network reaches a stable state, i.e., an attractor. Ideally, this attractor would be the stored pattern that one wanted to recover, however, the network could reach a spurious attractor which in general is not desired (Hopfield et al., 1983). Using a degraded version of the stored patterns is possible because each attractor has a basin of attraction which is a region surrounding the attractor in state space and whose dynamic converge into such attractor over time. So, an incomplete or noisy pattern that lies inside the basin of attraction of the correct pattern will, when updating the states, converge into the appropriate stored pattern.

On-line learning rules are suitable when the training patterns vary over the time. The incremental Hebb rule is an instance of this kind of rule. Some complex properties arise when Hebbian learning is applied in a Hopfield network. Vico and Jerez (2003) summarize these properties as follows: “(1) a generalization process takes place when patterns belonging to the same class (noisy versions of a prototype) are presented, activating the same attractor, (2) classes with low or no correlation are represented with different attractors, causing the reorganization of the synaptic matrix, and (3) new classes that correlate with previously stored classes involve a competitive-cooperative process among recalled attractors and new unstructured sets of network neurons, resulting in the formation of new attractors, while the old representations are interfered to some extent” (p. 13).

On the other hand, optimization is based on mapping a constraint satisfaction problem into the network topology. The Hopfield neural network has an energy function which was used to prove the stability of the network under the condition of symmetric and no self-recurrent connections. These conditions guarantee that the network always reaches a stable state, i.e., an attractor, regardless the initial network state. The energy decreases until reaching this state. Convergence into attractors is based on asynchronous state updates and, in the case of the Hopfield models with continuous states, a strictly monotone increasing activation function. The convergence of the network into an attractor represents the way that the activity of the components is coordinated so as to satisfy the constraints imposed by the weights. The energy function must have the same form as the function to be optimized, so that minima of the former are also minima of the latter. In this way, the value of the energy is interpreted as the amount of constraints that remain unsatisfied, that is, the greater the energy the less constraints have been satisfied. Thus, attractors are possible solutions of the mapped problem and minima of the energy function. Usually, the network is allowed to converge from different initial conditions so as to find many different attractors with the hope to thereby get the best possible solution. Nevertheless, finding the best solutions is challenging, because the landscape of attractors of a complex optimization problem is typically dominated by suboptimal solutions.

A lot of research has been done on associative memory and optimization in Hopfield networks. Many authors have noticed different problems in both applications. However, as far as this work is concerned, only problems related to optimization are most relevant and are therefore briefly presented and discussed. Joya et al. (1997) point out the three most common problems found in many published papers:

(1) “Many described applications do not coherently make a correspondence between the network dynamics and the energy function associated to that network. A common situation is using an analog neuron network with a continuous dynamics and associating it an energy function corresponding to a discrete neuron network” (p. 557). Solutions of the optimization problems solved by the Hopfield network are discrete such that the expected solutions are in the corner of the state space hypercube. However, in Hopfield models with continuous activation function an extra term is added to the energy function. This term is often called the integral term and causes the presence of attractors inside the hypercube. Therefore, the asymptotically stable states are in the neighborhood of the hypercube corners (Joya et al., 1997). The aim of the strategies for trying to avoid this problem is to make negligible the effect of the integral term. The most popular approach is using the continuous-time Hopfield model in the high-gain region. In this region, the gain of the activation function provokes an increment in the slope of the sigmoid function such that it resembles the sign function used in the discrete-state Hopfield models (Hopfield, 1984).

(2) “The energy function is forced to decrease only if the network evolves according to its dynamical equations. If these equations are continuous (differential equations), they can not be strictly represented by means of a computer simulation. That is, the simulation implies the discretization of these equations (difference equations), so that the bigger is the simulation step the more different are the real and the theoretical network behavior” (p. 557). This problem can be solved by ensuring numerical stability of the integration method. For example, the Forward Euler method is stable if the step is less than twice the smallest time-constant of the network (Blynel and Floreano, 2002). However, if there exist self-recurrent connections, the Euler method might increase occasionally the energy when the network is converging into an attractor (Feng and Douligeris, 2001).

(3a) “The energy function of a Hopfield network has many local minima. Consequently, the network probably will reach an equilibrium state that does not correspond to a problem solution” (p. 557). It is important to clarify the concept of stability in Hopfield networks. Usually, the network is said to be Lyapunov stable if the conditions of symmetric and no self-recurrent weights, and of a strictly monotone activation function are satisfied. However, Liao et al. (2007) point out that Lyapunov stability and Hopfield stability are different. The network is said to be Hopfield stable if the set of equilibria of the dynamical system which describes the network is attractive. The set of equilibria is said to be attractive if for any initial condition sufficiently near some equilibria, the solution of the system tends asymptotically to such element of the set of equilibria. Then, the energy function method only guarantees that a solution tends to an equilibrium, but this state may be unstable in the sense of Lyapunov. Also, this method can not answer if a specific equilibrium is stable or attractive. Furthermore, attraction of the set of equilibria is not equivalent to the attraction of every equilibrium. Consequently, the network might not be capable of reaching superior optima.

(3b) “The search of evolution strategies to move the network out of local minima and take it to a global minimum is a main task in this field”. Several efforts have been made for addressing this issue depending on the features of the problem required to be solved (Smith, 1999). Apart from all the research done on the deterministic Hopfield models, the approaches for avoiding local minima are mainly based on using heuristic methods together with the network (Potvin and Smith, 2003) or applying its stochastic counterpart, namely the Boltzmann machine (Kirkpatrick et al., 1983).

Recently, Watson et al. (2011b) proposed an algorithm for enhancing the ability of the discrete-time discrete-state Hopfield neural network to find configurations that minimize constraints in tension and therefore to minimize energy. The process takes advantage of both the network's power of learning patterns and its ability of minimizing conflicts between states during the convergence into a stable state. At first sight the operation of these applications seems incompatible. On the one hand, the weights change over time when the network is learning patterns. On the other hand, the weights are maintained for solving an optimization problem because the attractors, i.e., possible solutions, are defined by them. Despite this, Watson and colleagues developed a process based on allowing the neural network to form an associative memory of its own attractors—hence permitting it to learn its own previous dynamic patterns in an unsupervised manner—and thereby optimizing the network's capacity for optimization of constraint satisfaction.

## A review of self-optimization in neural networks

### The basic self-optimization mechanism

The self-optimization framework was originally developed for being applied in discrete-time discrete-state Hopfield neural network with symmetric weights and self-recurrent connections set to either 0 or 1. This model is updated according to Equation (1) in Table 1. The iterative algorithm consists in the following steps:

S1) the neuron states are randomized,
S2) the network is allowed to converge from this random state configuration to an attractor – this is called the “relaxation” of the network and lasts $\hat{t}$ time steps, and
S3) the network learns its current configuration, i.e., the attractor reached at convergence, according to the Hebbian rule shown in Equation (2).

**Table 1:** Comparison of two implementations of self-optimization.

**Reference**	Watson et al., 2011b		Zarco and Froese, 2018	
Update rule	$s_{i}\left(t+1\right)=\theta_{HT}\;\left[\overset{N}{\underset{j}{\sum }}\omega_{ij}\left(t\right)s_{j}\left(t\right)\right]$	(1)	$s_{i}\left(t+1\right)=\;\sigma \left[\overset{N}{\underset{j}{\sum }}\omega_{ij}\left(t\right)s_{j}\left(t\right)\right]$	(5)
Learning rule	ω_*ij*_(*t*+1) = ω_*ij*_(*t*)+δ*s*_*i*_(*t*)*s*_*j*_(*t*)	(2)	ω_*ij*_(*t*+1) = ω_*ij*_(*t*)+δ*s*_*i*_(*t*)*s*_*j*_(*t*)	(6)
Energy function	$E=\;-\frac{1}{2}\overset{N}{\underset{i,j}{\sum }}\omega_{ij}\left(t\right)s_{i}\left(t\right)s_{j}\left(t\right)$	(3)	$E=\;-\frac{1}{2}\overset{N}{\underset{i,j}{\sum }}\omega_{ij}\left(t\right)s_{i}\left(t\right)s_{j}\left(t\right)+\overset{N}{\underset{i}{\sum }}\int_{0}^{s_{i}\left(t\right)}\sigma^{-1}\left(x\right)dx$	(7)
Original energy function	$E_{0}=\;-\sum_{i,j}^{N}\alpha_{ij}\left(t\right)s_{i}\left(t\right)s_{j}\left(t\right)\;$	(4)	$E_{0}=\;-\frac{1}{2}\overset{N}{\underset{i,j}{\sum }}\alpha_{ij}\left(t\right)s_{i}\left(t\right)s_{j}\left(t\right)+\overset{N}{\underset{i}{\sum }}\int_{0}^{s_{i}\left(t\right)}\sigma^{-1}\left(x\right)dx$	(8)

*N is the number of neurons, s_i_ is the state of the neuron i, ω_ij_ is the weight between neuron i and j, θ_HT_ is a Heaviside threshold function, σ is a bipolar sigmoid function, and δ is the learning rate*.

The network is usually allowed to relax using a fixed $\hat{t}$ such that the network consistently reaches an attractor. Notice that the time steps for reaching an attractor, t^*^, is smaller than $\hat{t}$. Depending on how small t^*^ is compared to $\hat{t}$, learning could be done either at the end of the relaxation or at every time-step. Ideally, the iterative process stops when a single global attractor remains in the state space.

The energy function in Equation (3) is associated with the discrete-time discrete-state Hopfield model. Watson and colleagues use the so-called original energy function shown in Equation (4) to compute the degree to which a state configuration resolves the original constraints. This function uses the original weights of the problem and the state configuration at the end of the relaxation. Both energy functions have the same quadratic form, the only difference is the constant in the function proposed by Hopfield. The lack of this constant does not affect the function of illustrating the amount of satisfied constraints.

There exist three conditions for the iterative algorithm to work:

C1) the initial dynamic of the system exhibits multiple attractors,
C2) the system configurations are repeatedly relaxed from different initial conditions such that the system samples many different attractors on a timescale where connections change slowly, and
C3) the system spends most of its time at attractors.

The condition C3 is mandatory if learning is applied at every time-step, but such condition could be relaxed if learning is done at the end of the relaxations. Presumably, reinforcing states near an attractor should be sufficient since they would have enough sub-pattern in common with such attractor, however a thorough investigation has never been done.

As a consequence of these conditions, there are two practical requirements:

R1) the learning rate must be small so that the system reaches a wide number of attractors and poor local optima are not reinforced;
R2.1) if learning is applied at the end of the relaxation, $t^{*}<\hat{t}$ so that the system reaches the stable state before reinforcing it; and
R2.2) if learning is applied at every time-step, $t^{*}\ll \;\hat{t}\;$so that the system spend little time at the transient dynamic and the stable state is properly reinforced during the relaxation time.

Due to $t^{*}\ll \;\hat{t},$ learning at every time-step is possible since a learning rate used at the end of the relaxation could be divided by $\hat{t}$ such that the network can imprint reliably its current configuration.

According to Watson et al. (2011c), the self-optimization process is grounded in three properties of the discrete-time discrete-state Hopfield model:

P1) *Associative memory of attractors*. In general, it is more likely that the network converges into locally optimal solutions to the problem just by relaxation. Reinforcing its own attractors, i.e., forming an associative memory of its own attractors, has the effect of increasing the basin of attraction of these locally optimal attractors.
P2) *Inverse correlation between energy and size of basins of attraction* (Kryzhanovsky and Kryzhanovsky, 2008). It has been proven that low energy attractors tend to have larger basins of attraction. Therefore, when the network is reinforcing its attractors, the network tends to converge more into low-energy attractors more often than higher-energy attractors.
P3) *Generalization* based on a combination of properties P1 and P2.

There are two kinds of generalization depending on the structure of the problem (Watson et al., 2009, 2011a):

G1) *Noise removal in unstructured problems* (Branchtein and Arenzon, 1992). In these problems, which can be defined by random connectivity, there are globally optimal attractors which overlap on average equally with all the locally optimal attractors in their vicinities. The former are located in the center mass (or near it) of the latter. The network tends to converge in locally optimal attractors in the vicinity of a global optimum. Thus, the reinforcing of the former leads to the enlargement of the basin of attraction of the latter.
G2) *Spurious attractors in structured problems* (Ju-Seog et al., 1992). In these problems, which can be defined by modular connectivity, there are large semi-independent modules that are separated by energy barriers. The attractor in modules have sub-pattern in common with other attractors in other modules. The network reaches easily modules with locally optimal attractors. The reinforcing of these attractor could create spurious attractors which have sub-patterns in common with learned attractors. Spurious attractors could represent better possible solutions of the original optimization problem. Thus, the reinforcing of local attractors leads to the enlargement of the basin of attraction of spurious attractors.

In both cases, either generalization by noise removal or spurious attractors, the enlargement of the basin of attraction of better attractors leads to these outcompeting only locally optimal attractors. These new representations can be used as attractors to be reinforced by convergence or, if they are distant, their basins of attraction can continue being enlarged. In general, the reinforcement of locally optimal attractors can reinforce globally optimal attractors since, at least in structure problem spaces, they have several sub-patterns in common, even if some of them have not been reached previously.

Step S1 of the process, i.e., the randomization of the initial state configuration, makes possible the exploration of the state space in order to converge on and reinforce many different attractors. Meanwhile, Hebbian learning makes possible the exploitation of the correlation between locally and globally optimal attractors. Learning does not change the configuration of attractors in the state space, but makes possible the enlargement of the best basins of attraction, and therefore leads to a competition between attractors.

### Variations in neural network topology

Table 2 shows the types of constraints used to define the weights in different works in which the self-optimization process was studied by Watson and colleagues. Symmetric weights with non-negative self-recurrent connections is a good assumption because the convergence of a discrete-time discrete-state Hopfield network into attractors, regardless the initial condition, can be guaranteed (Hopfield, 1982). The constraint of symmetric weights would be easy to relax in this model given the fact that an asymmetrical weight matrix, *W*, could be changed for its symmetric version, $\frac{\left(W+W^{T}\right)}{2}$, without changing the value of the energy (Vidyasagar, 1998). According to Xu et al. (1996), the constraint of no self-recurrent weights can be avoided as long as $\omega_{ii}\geq \left(\frac{1}{2}\right)\overset{N}{\underset{j=1,j\neq i}{\sum }}|\omega_{ij}-\omega_{ji}|$. The energy is called regular if this condition is met, however, the set of minima of the energy function is a subset of the set of all stable states of the network. On the other hand, if the condition $\omega_{ii}\leq -\left(\frac{1}{2}\right)\overset{N}{\underset{j=1,j\neq i}{\sum }}|\omega_{ij}-\omega_{ji}|$ is met, the energy is called normal, and the set of all stable states of the network is a subset of the set of minima of the energy function.

**Table 2:** Types of condition used in the research done by Watson and colleagues.

**References**	**Condition**	**Definition (α_*ij*_≡ω_*ij*_(*t* = 0))**
Watson et al., 2009	Spatial	$\alpha_{ij}\in \left(0,\;e^{-d}\right)\text{ where }d=\text{mod}\left(|1-j|,\;N\right)$
	Modular	$If\;\left\lfloor \frac{i}{k}\right\rfloor =\left\lfloor \frac{j}{k}\right\rfloor \text{ and }i\neq j,\;|\alpha_{ij}|=1$ Otherwise, α_*ij*_ = *p*, with *p* < 0.01
Watson et al., 2011b	Random	If *i* = *j*, α_*ij*_ = 0 Otherwise α_*ij*_ ∈ {−1, 1}
	Modular	$If\;\left\lfloor \frac{i}{k}\right\rfloor =\lfloor \frac{j}{k}\lfloor \text{ and }i\neq j,\;|\alpha_{ij}|=1$ Otherwise, α_*ij*_ = 0.01
Watson et al., 2011a	Random Sparse	α_*ij*_ ∈ {−1, 0, 1}$\text{the density of nonzero connections is }\frac{m}{N}$
	Modular	$If\;\left\lfloor \frac{i}{k}\right\rfloor =\left\lfloor \frac{j}{k}\right\rfloor \text{ and }i\neq j,\;|\alpha_{ij}|=1$ Otherwise, α_*ij*_ = 0.01
Watson et al., 2011c	Random (RC)	If *i* = *j*, α_*ij*_ = 1 Otherwise, α_*ij*_ = {−0.01, 0.01}
	Modular	$\alpha_{ij}=\text{RC}\left(\lfloor \frac{i}{k},\;\frac{j}{k}\rfloor \right)$

*N is the number of neurons, k is the number of modules, and $\frac{m}{N}$ means m dependences per node on average. In the random constraints condition, binary connection weights {−1, 1} are chosen with equal probability; in the modular constraints condition, k = 5 and α_ij_ > 0 with probability 0.8; and, in the random spatial constraints, m = 8 and {−1, 1} are chosen with equal probability*.

### Further variations and extensions

Table 3 presents further studies that explored the general conditions under which self-optimization can take place. Moreover, the underlying principle of the self-optimization process has been used to model the evolution of gene regulation networks where changes in the activation level of the genes were computed according to the dynamic of a discretized CTRNN (Watson et al., 2010). However, in that work gains and time constants were fixed values, and there were no bias terms. Also, the energy function had the quadratic form as that in Equation (4) because they were measuring epistasis. Thus, the aim of that work was somewhat different from our goal of generalizing the self-optimization process to the kind of CTRNNs used in evolutionary robotics. Moreover, Beer (1995) demonstrated the complexity of the CTRNN state space, i.e., the emergence of several different limit sets, of one- and two-neuron recurrent CTRNNs, when the parameters are changed. Therefore, we still face the challenge of extending the self-optimization framework so that it can be applied to CTRNNs with arbitrary gains, arbitrary time constants, and asymmetric and self-recurrent connections.

**Table 3:** Activation function, states range, and weights range used in work that explored the general conditions under which self-optimization takes place in complex networks.

**References**	**Activation function**	**States**	**Weights**
Watson et al., 2010	Sigmoid function (tanh(*x*/10))	[−1, 1]	ω_*ij*_ is not bounded
Watson et al., 2011a	Heaviside threshold function	{−1, 1}	−1 ≤ ω_*ij*_ ≤ 1
Woodward et al., 2015	Saturated linear function	[0, 1]	−1 ≤ ω_*ij*_ ≤ 1
Zarco and Froese, 2018	Sigmoid function ($\frac{2}{1+e^{-x}}-1$)	[−1, 1]	−1 ≤ ω_*ij*_ ≤ 1

## Methods

In this work, a fully connected CTRNN is used. The network consists of *N* nodes and it is updated asynchronously according to the following equation:

(9) $$\begin{array}{r} \tau_{i}{ṡ}_{i}=\;-s_{i}+\overset{N}{\underset{j=1}{\sum }}\omega_{ji}\sigma \left(g_{j}\left(s_{j}+\theta_{j}\right)\right) \end{array}$$

where *s*_*i*_ is the activation of node *i*, ω_*ji*_ is the strength of a connection from node *j* to node *i*, τ_*i*_ is the activity decay constant (or time constant), *g*_*i*_ is the gain of the sigmoid function, θ_*i*_ is the following bias term (Golos et al., 2016):

(10) $$\begin{array}{r} \theta_{i}\;=\;-\frac{1}{2}\overset{N}{\underset{j=1}{\sum }}\omega_{ij} \end{array}$$

and σ is the following sigmoid activation function (Hoinville et al., 2011):

(11) $$\begin{array}{r} \sigma \left(x\right)=\;\frac{2}{1+e^{-x}}-1 \end{array}$$

*V*_*j*_(*t*) = σ(*g*_*j*_(*s*_*j*_(*t*)+θ_*j*_)) is called the output of the neuron *j*, and we will refer to 〈*V*_1_, …, *V*_*N*_〉 as the output state space hypercube.

Golos et al. (2016) demonstrated the emergence of multiple fixed-point attractors using a 998 node continuous-time continuous-state Hopfield model in the high-gain region, with symmetric weights and the bias term computed according to Equation (10). In this work, this equation is an educated guess because it is used in a small non-restricted Hopfield model, that is, with asymmetric weights and outside the high-gain region. The authors cannot guarantee that the equation always generates enough attractors given the amount of parameters that could be changed in the network.

The steps of the self-optimization iterative algorithm for CTRNNs are the following:

S1) node activations, *s*_*i*_, are drawn from a uniform random distribution limited by a lower and upper bound,
S2) the network is relaxed, during ť units of time, from this random configuration, and
S3) the weights are updated at the end of the relaxation according to the following equation:

(12) $$\begin{array}{r} \omega_{ij}\left(t+1\right)=\omega_{ij}\left(t\right)+\delta V_{i}\left(t\right)V_{j}\left(t\right) \end{array}$$

where δ is a learning rate and each ω_*ij*_ is restricted to be between [−1, 1]. Ideally, the iterative process continues until the network converges into a single attractor for any initial state configuration. The lower and upper bounds are not limited to −1 and 1, respectively, as in previous works because, depending on the values of the parameters, a broader range could be necessary for exploring the state space.

Due to the non-restricted connections and parameters, neither the fast convergence into an attractor nor the convergence into an attractor in every relaxation period is guaranteed. Therefore, Hebbian learning is applied at the end of the relaxation for two reasons: (1) given the possibility of a long transient dynamic, it is more likely that the network would have reached an attractor at the end of that time, thus reinforcing such attractor or, at least, (2) the network would have reached a basin of attraction, hence reinforcing a point in the neighborhood of the attractor which probably has enough sub-patterns in common with such attractor. However, it is also possible that there exist other kinds of limit sets but, since Hebbian learning tends to reduce the complexity of the state space (Siri et al., 2008), we do not consider them further here.

For the experiments, the network had 30 nodes, time constants were randomized between [1, 10] using a uniform distribution, gains were randomized between [10, 20] using a uniform distribution, bias terms were computed using the Equation (10), ť = 500 units of time, and the Equation (9) was integrated using the forward Euler integration method with a step size of 0.1. The connection matrix was initialized using 100 different weight configuration per each type of condition: symmetric random condition (SR), asymmetric random condition (AR), symmetric modular condition (SM), and asymmetric modular condition (AM). Table 4 shows the type of conditions used in the experiments of this work. In the symmetric random condition, the values of $\alpha_{ij}^{SR}$ were chosen using a uniform random distribution; in the symmetric modular condition, *k* = 3 and $\alpha_{ij}^{SM}>0$ with probability 0.8; in the asymmetric random condition, *q*>0 with probability 0.5 and the asymmetric connections are defined adding a value from a uniform random distribution to *q*; and, in the asymmetric modular condition, asymmetric connections are defined adding a value from a uniform random distribution to a symmetric modular matrix.

**Table 4:** Types of conditions used to define weight matrices in this work, where *k* is the number of modules.

**Condition**	**Definition **(**α_ij_≡ω_ij_**(*t* = 0))****
Symmetric Random (SR)	$\text{If }i=j,\;\alpha_{ij}^{SR}\in \left[-1,1\right]$ $\text{Otherwise }\alpha_{ij}^{SR}\in \left[-0.1,0.1\right]$
Asymmetric Random (AR)	$\text{If }i=j,\;\alpha_{ij}^{AR}{=\alpha }_{ij}^{SR}$ $\alpha_{ij}^{AR}=q+\left[-0.1,\;0.1\right]\;with\;|q|=0.1$
Symmetric Modular (SM)	$\text{If }\left\lfloor \frac{i}{k}\right\rfloor =\left\lfloor \frac{j}{k}\right\rfloor ,\;|\alpha_{ij}^{SM}|=1$ $\text{Otherwise }|\alpha_{ij}^{SM}|=0.01$
Asymmetric Modular (AM)	$If\;i=j,\;\alpha_{ij}^{AM}=\alpha_{ij}^{SM}$ ${\text{ Otherwise }\alpha_{ij}^{AM}=\alpha }_{ij}^{SM}+\left[-0.01,\;0.01\right]\;$

Given that it is difficult to determine a common learning rate for the four conditions, the learning rates was found empirically such that δ = 0.001 for the symmetric cases, and δ = 0.0005 for the asymmetric ones. Both learning rates were estimated through many trials, setting a specific number of relaxations during self-optimization, to avoid suboptimal convergence. In other words, the learning rates were chosen to increase the chance that the networks reinforce enough attractors such that the best solutions outcompete suboptimal ones. If the learning rate for the symmetric cases were used for all the experiments, the asymmetric networks would converge into suboptimal attractors. On the contrary, if the learning rate for the asymmetric cases were used, the symmetric networks would probably require to increase the number of relaxations in order for the network to converge into a single attractor.

CTRNNs have been used before to solve specific constraint satisfaction problems using a generalized energy function (Ettaouil et al., 2013; Haddouch et al., 2013). In that approach, an energy function is defined by taking into account the objective function and a function to penalize the violation of task-correlated constraints. After that, weights are defined according to this function and a parameter setting procedure is applied such that each attractor corresponds to a solution of the problem. However, our approach is more general because weights and parameters can be arbitrarily set, even setting asymmetric connections, and therefore we cannot guarantee the one-to-one correspondence between attractors and solutions to the original problem. Moreover, Zarco and Froese (2018) showed that, in the case of discrete-time continuous-state Hopfield neural networks, there is no correlation between the energy and the number of satisfied constraints. Accordingly, they argued that it is better to use the number of satisfied constraints directly rather than the energy function. Thus, in the current work the number of satisfied constraints was measured considering that a constraint is satisfied in a CTRNN if α_*ij*_*V*_*i*_*V*_*j*_ > 0, where *V*_*i*_ ∈ [−1, 1].

## Results

The experiments consisted in three stages per each type of condition. First, an initial weight configuration was set and the network relaxed from 100 different random initial states configurations. This constitutes the “before-learning” stage. Then, the self-optimization process was applied using 1,000 relaxations. Finally, the network was relaxed from 100 different random initial states configurations using the modified weight configuration obtained by the self-optimization process. This constitutes the “after-learning” stage. The amount of satisfied constraints was measured at the end of each relaxation in the “before-learning” and the “after-learning” stages. This procedure was repeated 100 times per each one of the 100 weight configurations using a different random number seed.

To measure the success of the self-optimization process, we decided to run a *t*-test per each repetition, instead of considering the whole data set of satisfied constraints, for two reasons: (a) as long as there is some effect size or, to put it differently, the effect size is not zero, statistical significance will be almost always demonstrated with a sufficiently large sample size (Sullivan and Feinn, 2012); and (b) each repetition can be considered as an individual experiment given that each weight configuration is tested many times in the “before-learning” and the “after-learning” stages using different random number seeds. Thus, the computation of the *p*-values reported in this work consisted in two steps. First, we calculated the *p*-values of each one of the 100 repetitions per each one of the 100 different weight configuration. Then, we calculated the mean of those *p*-values.

### Random constraints conditions

**Figure 1:**
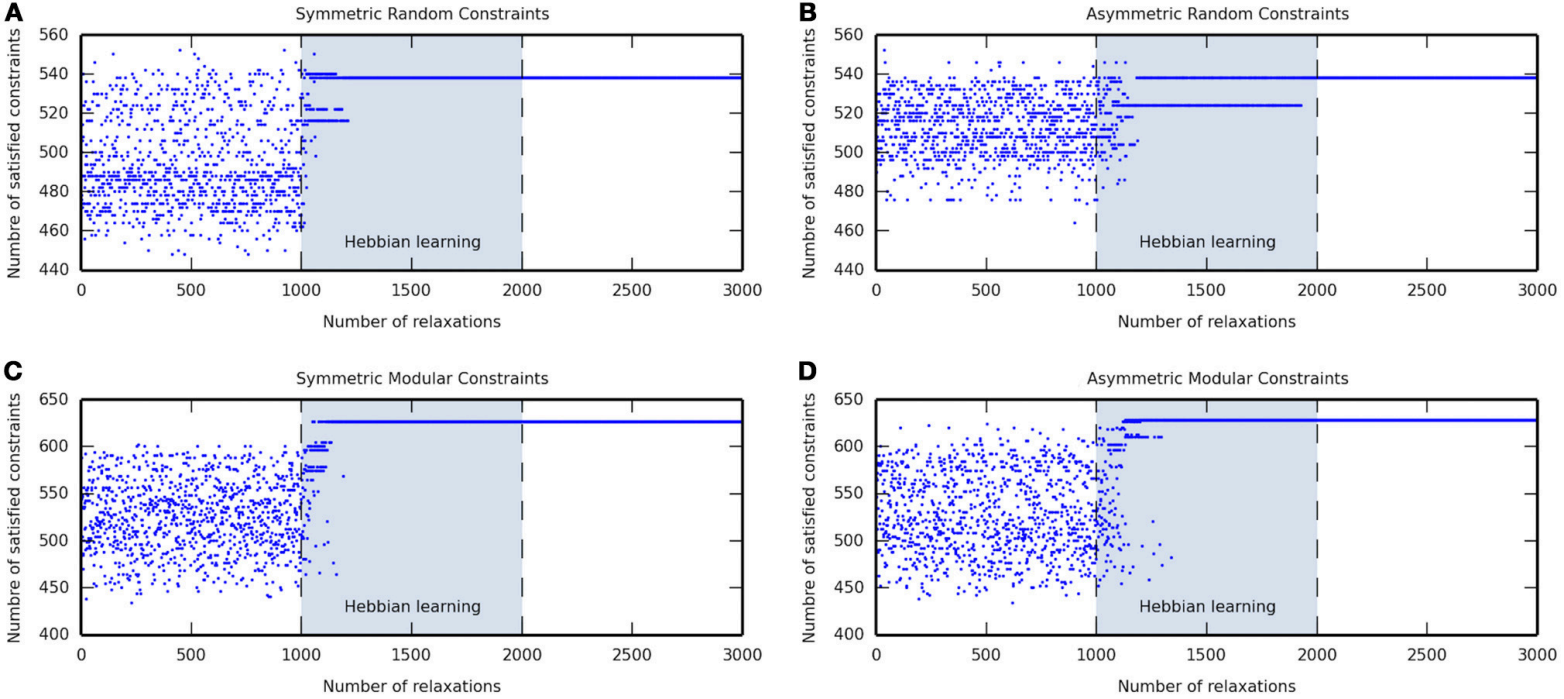
Examples of the self-optimization process in four different constraints conditions. Each figure shows the attractor states visited before learning (relaxations 1–1000), during the self-optimization process (relaxations 1001–2000), and after learning (relaxations 2001–3000). Note that neural network convergence after activations are set to arbitrary values, and the number of satisfied constraints are counted after the completion of a relaxation.

Figures 1A,B show the attractor states reached by a network with symmetric and asymmetric random connections, respectively, in the before-learning phase (relaxations 1–1000), during the self-optimization process (relaxations 1001–2000), and in the after-learning phase (relaxations 2001–3000). In the before-learning phase, the network was allowed to relax and it converged into many different attractors; overall, a great number of suboptimal attractors were visited, but just a few attractors that could be considered as good solutions were reached. During the learning phase, the self-optimization process shifted the distribution to these better attractors, but without significantly exceeding the attractors that were accessible before. As can be seen, the network converged into better solutions as the number of relaxations increase until one single attractor remained. Finally, in the after-learning phase, where the network was allowed to relax without still applying learning, this better attractor was reached every relaxation because it is either the only attractor in the state space or at least the more likely to be reached.

**Figure 2:**
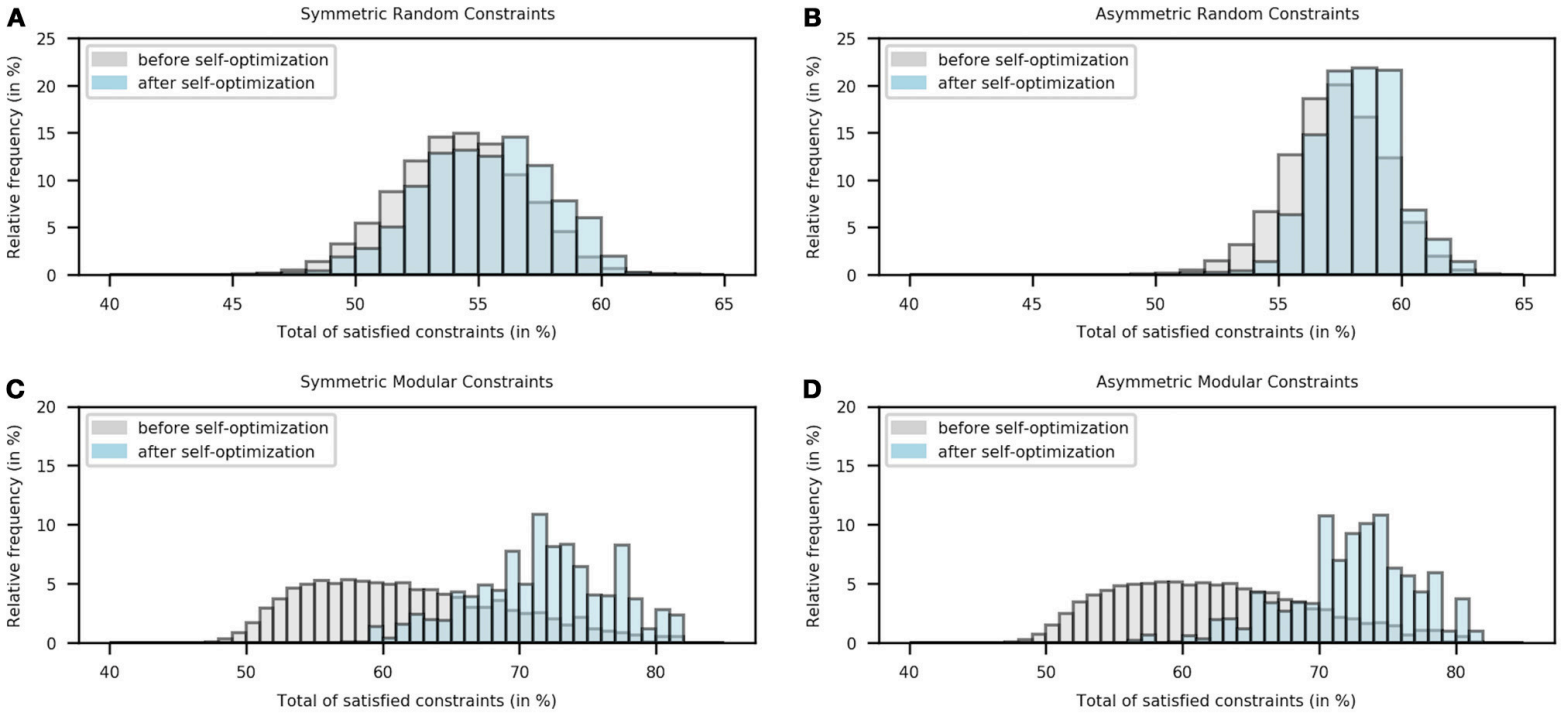
Histogram of the number of satisfied constraints in the “before learning” stage (gray) and the “after-learning” stage (blue) for four different constraints conditions. Note that the relative frequency was calculated following 1000000 relaxations.

Figures 2A,B show the distribution of the number of satisfied constraints in symmetric and asymmetric connections for random constraints conditions. The total number of connections in a 30-node network is 900. On average, 54.1835% (487.6517 connections) and 57.2478% (515.2306 connections) constraints were satisfied before self-optimization in the symmetric and asymmetric case respectively; while the corresponding numbers of satisfied constraints after self-optimization were 55.2443% (497.1987 connections) and 58.1547% (523.3925 connections). This difference turned out to be little significant given the *p*-value of 0.0640 in the symmetric case and the *p*-value of 0.0705 in the asymmetric one.

### Modular constraints conditions

Figures 1C,D show the attractor states reached by a network with symmetric and asymmetric modular connections, respectively, in the before-learning phase (relaxations 1–1000), during the self-optimization process (relaxations 1001–2000), and in the after-learning phase (relaxations 2001–3000). As in the random constraints conditions, in the before-learning phase, the network converged into many different attractors of which many of them were suboptimal and only few ones could be taken as good solutions. Nevertheless, in the modular cases, the self-optimization produced a significant shift of the distribution, such that after the process the network was able to access solutions that were not accessible before. The network converged into better attractors as the number of relaxations increase until one single attractor remained which usually could not be reached before. Finally, the network converged into this attractor every relaxation in the after-learning phase because it became either the only attractor in the state space or at least the more likely to be reached.

Figures 2C,D show the distribution of the number of satisfied constraints in symmetric and asymmetric connections for modular constraints conditions. On average, 61.8042% (556.2378 connections) and 62.1478% (559.3308 connections) constraints were satisfied before self-optimization in the symmetric and asymmetric case respectively; while the corresponding numbers of satisfied constraints after self-optimization were 71.7320% (645.5885 connections) and 72.3285% (650.9570 connections). This difference turned out to be very significant given the *p*-value of 1.1235 × 10^−5^ in the symmetric case and the *p*-value of 4.58800 × 10^−6^ in the asymmetric one.

## Discussion

The results in Figure 1 show that the self-optimization process works properly since it increases the basins of attraction of better attractors, as measured by their ability to satisfy interneuron constraints. As can be seen during the self-optimization phase (relaxations 1001-2000), it becomes increasingly likely that a CTRNN converges on attractors which satisfy more constraints, compared with those that the network can reach before reinforcing its visited attractor state configurations. The reinforced attractors start outcompeting each other over time. This results in, ideally, the existence of just one optimal attractor in the activation state space, which is near or in some corner of the hypercube. As stated by Watson et al. (2011b) for the case of discrete-time discrete-state Hopfield networks, the self-optimization process in CTRNNs works much better on modular or structured problems because it exploits the correlation between local and global optima. This is demonstrated by the fact that after self-optimization (relaxations 2001–3000), modular networks tend to converge on solutions that are better at satisfying constraints than any solution they were able to find before self-optimization (relaxations 1–1000).

Figure 2 shows that the results found in single examples are also statistically true. As can be seen, there exist small differences between the distributions in Figures 2A,B. The differences between the percentages of satisfied random constraints before and after self-optimization are 1.0608 and 0.9070% when symmetric and asymmetric connections are set, respectively. The little-significant *p*-values (0.0640 and 0.0705) of these cases are then explained by the slight increase in the number of satisfied constraints due to the self-optimization process. These results were expected given that the process is not able to exploit the regularities of the connections.

On the other hand, there exist big differences between the distributions in Figures 2C,D. The differences between the percentages of satisfied modular constraints before and after self-optimization are 9.9278 and 10.1807% when symmetric and asymmetric connections are set, respectively. Thus, the very-significant *p*-values (1.1235 × 10^−5^ and 4.58800 × 10^−6^) of these cases are explained by the meaningful rise in the number of satisfied constraints due to learning. In this case, the self-optimization process is able to exploit the correlation between local and global optima as stated before. This is not to say that the experiment of the random case failed as can suggest a *p*-value lower than 0.05; on the contrary, the self-optimization process works on unstructured problems but the results are not as good as in the modular case.

We have shown that the self-optimization works for CTRNNs with self-recurrent and either symmetric or asymmetric connections. The results demonstrate that the self-optimization process has the potential for a broader range of applications than previously thought.

## Future work

As we mentioned in the introduction, a particularly suitable area for future research could be the “world-involving” type of scenarios generated by means of an evolutionary robotics approach: when an agent is evolved to exhibit some kind of adaptive behavior, it often turns out that its CTRNN controller only exhibits a single attractor and yet is still capable of rich dynamics because its attractor landscape is continuously modulated by the agent's embodied coupling with the environment in a task-relevant manner. In this type of scenario, the evolutionary optimization process basically results in a CTRNN structure that makes the agent ready to be interactively guided by the world. These findings go against the perspective of classical cognitive science, according to which the internal dynamics of the agent's brain carry all of the weight of cognition, but they make sense from a dynamical perspective that treats behavior and cognition as a relational property of a brain-body-environment system (Beer, 2000).

Now that we know that the self-optimization process works for decoupled CTRNNs, a challenge for future research is to determine how it could be employed instead of an evolutionary process to generate the appropriate structure while coupled to the agent's body and environment. If this could be successfully implemented, the advantage would be that the agent could learn the best world-involving solutions during its lifetime, and also readjust its internal structure as the demands of the task change over time.

One key challenge lies in making sure that the optimal attractor that is going to be reinforced by the self-optimization process is one that is most relevant for the task. Starting the process with a random CTRNN, as we have done here to show its generality, could for example just lead to an agent that does precisely nothing or just moves in one direction. This problem of how to connect the internal plastic mechanism with the desired behavior in a meaningful manner is already well known from the homeostatic adaptation mechanism literature (Iizuka and Di Paolo, 2008). One way of addressing it would be to evolve the starting configuration of the CTRNN, along with the parameters of the self-optimization process, such that the agent tends to improve its performance over trials. Perhaps this starting configuration could even be evolved to be suitable for self-optimization of distinct adaptive behaviors, for example depending on which type of trial it finds itself in, similar to what has already been achieved with non-plastic CTRNN controllers (Izquierdo and Buhrmann, 2008).

At this point it is not yet clear how explicit one needs to make the self-optimization process for it to evolve properly. Parameters that could be fine-tuned by artificial evolution include the learning rules, learning rates, reset mechanism, and number of relaxations. Alternatively, it could be sufficient to simply evaluate fitness of the solutions in terms of their relative improvement of performance during a trial, similar to what has been achieved for decoupled CTRNNs evolved to exhibit ultrastability (Izquierdo et al., 2013). That work has demonstrated that a CTRNN can indeed be evolved to iterate through a number of different attractors, and to do so in a context-sensitive manner, by temporarily passing through a metastable regime. In general, we expect that making the self-optimization process more explicit will facilitate the evolution of a workable solution, but it may also preclude the evolution of more elegant implementations of the process.

An interesting open question is whether there will continue to be a role for decoupled dynamics even when the CTRNN is embodied in an agent. To find out what works best it may be useful to explicitly give the agent the ability to temporarily switch on and off its coupling with the environment, as has been explored in a different context by Iizuka and Ikegami (2004). If there is some penalty for poor performance, it is likely that agents will evolve to self-optimize during short periods of sensorimotor decoupling in order for the temporary randomization of neuronal activations not to lead to unwanted behavior. At the same time being decoupled will prevent sensory stimulation from interfering with the self-optimization process, thereby removing the formal problem of how to implement the process in a coupled CTRNN.

## Author contributions

All authors listed have made a substantial, direct and intellectual contribution to the work, and approved it for publication.

### Conflict of interest statement

The authors declare that the research was conducted in the absence of any commercial or financial relationships that could be construed as a potential conflict of interest.
